# Role of Homeodomain Leucine Zipper (HD-Zip) IV Transcription Factors in Plant Development and Plant Protection from Deleterious Environmental Factors

**DOI:** 10.3390/ijms14048122

**Published:** 2013-04-12

**Authors:** William Chew, Maria Hrmova, Sergiy Lopato

**Affiliations:** Australian Centre for Plant Functional Genomics, University of Adelaide, Waite Campus, Glen Osmond, South Australia 5064, Australia; E-Mails: william.chew@acpfg.com.au (W.C.); maria.hrmova@acpfg.com.au (M.H.)

**Keywords:** homeodomain, protein-DNA interactions, L1 cell layer, epidermis, cuticle

## Abstract

Homeobox genes comprise an important group of genes that are responsible for regulation of developmental processes. These genes determine cell differentiation and cell fate in all eukaryotic organisms, starting from the early stages of embryo development. Homeodomain leucine zipper (HD-Zip) transcription factors are unique to the plant kingdom. Members of the HD-Zip IV subfamily have a complex domain topology and can bind several *cis*-elements with overlapping sequences. Many of the reported HD-Zip IV genes were shown to be specifically or preferentially expressed in plant epidermal or sub-epidermal cells. HD-Zip IV TFs were found to be associated with differentiation and maintenance of outer cell layers, and regulation of lipid biosynthesis and transport. Insights about the role of these proteins in plant cuticle formation, and hence their possible involvement in plant protection from pathogens and abiotic stresses has just started to emerge. These roles make HD-Zip IV proteins an attractive tool for genetic engineering of crop plants. To this end, there is a need for in-depth studies to further clarify the function of each HD-Zip IV subfamily member in commercially important plant species.

## 1. Background

Transcription factors (TFs) are key components underlying mechanisms that control gene expression in all living organisms. They define phenotypic diversity and evolutionary adaptation of organisms [1,2]. TFs typically consist of at least two major domains. The first is a DNA-binding domain (BD) that recognises target DNA sequences and the second is a transcriptional activation domain (AD) that initiates transcription by interacting with general transcription factors [3].

Several TF families consist of proteins containing a DNA-binding domain known as the homeodomain (HD). The first HD-containing TF was identified in *Drosophila melanogaster*[4]. The homeotic gene encoding this TF was found to be responsible for the development of antennae at the position of the second leg pair of *Drosophila*[4]. Later, a large number of homeotic genes containing a 180 bp long homeobox (HB) were identified and their involvement in the development of *Drosophila* was demonstrated. Proteins with highly conserved HDs have been found in other animals, fungi and plants, indicating that these TFs play an important role in developmental pathways in species across different kingdoms [5–7]. Many HB-containing genes in mammals and *Drosophila* are homeotic genes that determine body part formation in specific positions. However, none of the plant HB-containing genes exert any such homeotic effect.

The first plant gene encoding a HD-containing protein, KNOTTED1, was isolated from maize (*Zea mays* L.) [8]. Since the identification of *KNOTTED1*, a large number of plant HB-containing TF genes have been isolated. Based on the presence of specific functional domains, plant HD-containing TFs were classified into six families: (1) homeodomain leucine zipper (HD-Zip); (2) plant homeodomain with a finger domain (PHD); (3) Bell domain (Bell); (4) zinc finger with homeodomain (ZF-HD); (5) Wuschel homeobox (WOX); and (6) Knotted homeobox (KNOX) [9]. A comprehensive classification of plant homeobox genes has been reported [10].

The HD-Zip family of TFs is unique to the plant kingdom, suggesting their involvement in developmental processes specific for plants [11,12]. The HD-Zip family consists of four subfamilies or classes. A common feature of all four subfamilies is the presence of a leucine zipper domain (Zip) adjacent to HD, which is important for homo- and hetero-dimerisation [9]. Members of the HD-Zip II subfamily can be distinguished from HD-Zip I subfamily proteins by the presence of a conserved “CPSCE” motif, which is situated downstream of the leucine zipper [6]. HD-Zip III and IV TFs contain a STeroidogenic Acute Regulatory protein-related lipid Transfer (START) domain and a conserved START-associated domain (HD-SAD), that are absent in HD-Zip I and II proteins [13,14]. It is of note that the START domains have never been associated to work in conjunction with DNA binding in mammalian systems, while the START domains have primarily been associated with the plant HD-Zip domains. Members of the HD-Zip III subfamily can be distinguished from HD-Zip IV subfamily members by an additional *C*-terminal MEKHLA domain [14]. Unlike other known HD proteins that are able to bind DNA sequence as monomers, HD-Zip TFs bind to DNA and consequently activate/repress downstream gene(s) only as dimers [15]. Several genes encoding members of HD-Zip proteins were shown to be involved in abiotic stress responses [16–22]. Class IV of HD-Zip TFs is involved in a number of vitally important processes, including the transcriptional control of epidermal and sub-epidermal cell fate, anthocyanin accumulation, lipid transport, and cuticle biosynthesis [23]. Involvement of HD-Zip IV TFs in mediating plant responses to drought have also been reported [17].

In this review we summarise existing knowledge on structural characteristics, expression patterns, potential interacting partners and target genes of HD-Zip IV TFs, as well as describe possible roles of the *HD-Zip IV* genes in plant development, defence and response to abiotic stresses. Potential applications of these genes in plant biotechnology will be discussed.

## 2. Structure of HD-Zip IV TFs and Function of Identified Domains

HD-Zip IV TFs consist of four conserved domains. A highly conserved HD domain contains 60 or 61 amino acid residues and is responsible for binding to a specific DNA sequence by forming a structure composed of three α-helices. It was shown that HD-Zip IV proteins from *Arabidopsis* preferentially bind an 11 bp-long palindromic sequence 5′-GCATT(A/T)AATGC-3′, which partly overlaps with the sequence of the L1 box (5′-TAAATG(C/T)A-3′) [24]. The L1 box is responsible for specific gene expression in the epidermal L1 layer [25]. This *cis*-element was identified in the promoter regions of several genes, including *HD-Zip IV* genes [25,26]. The L1 box was found in the promoter region of the *Arabidopsis PROTODERMAL FACTOR1* (*PDF1*) gene, which encodes a putative extracellular proline-rich protein with expression highly specific to the L1 cell layer of shoot apices and the protoderm of organ primordia. Expression of *PDF1* is regulated by an *Arabidopsis* HD-Zip IV TF known as *MERISTEM LAYER1* (*AtML1*), through binding of the L1 box in the promoter region of the *PDF1* gene [25]. The substitution of two base pairs (AT for GG) in the L1 box located in the *PDF1* promoter abolished binding of the AtML1 protein to the promoter in electrophoretic mobility shift assays (EMSA) [25]. However, HD-containing proteins often show a poor DNA-binding specificity in EMSA experiments and for strong DNA binding the HD-Zip IV proteins might require assistance from other TFs and/or modifying enzyme(s) [27]. Therefore, a final conclusion about *in vivo* activation of genes containing the L1 box in their promoters by AtML1 was not reached [25,27]. It is noteworthy that the plant L1 box sequence (5′-TAAATG(C/T)A-3′) resembles that of the target sequence (5′-TTAATGGCC-3′) of some homeotic proteins from *Drosophila*, such as Ultrabithorax [28].

**Figure 1:**
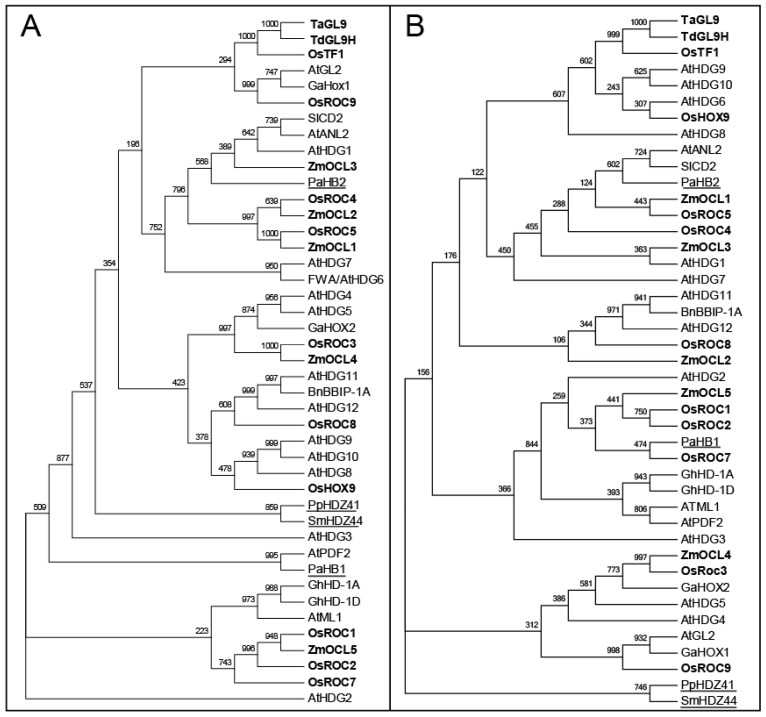
Unrooted phylogenetic tree of selected HD-Zip IV proteins. HD-Zip IV protein sequences were retrieved from the NCBI database and aligned with CLUSTALX [47]. Unrooted phylogenetic trees were constructed based on aligned protein sequences using the Neighbor-Joining algorithm [48] with a Bootstrap value of 1000 from CLUSTALX [47]. Species of origin are indicated by two-letter prefixes. The accession numbers of the published protein sequences used in the phylogenetic trees are listed in Table 1; unpublished BnBBIP-1A has Accession ABA54874. (**A**) Phylogenetic tree based on full-length amino acid sequences of 43 HD-Zip IV proteins; (**B**) Phylogenetic tree constructed using HDs of selected HD-Zip IV proteins (60–61 amino acid residues). The HD sequences that were included in the analyses were selected by a Simple Modular Architecture Research Tool (SMART) [49]. At, *Arabidopsis thaliana*; Pa, *Picea abies*; Ga, *Gossypium spp*; Gh, *Gossypium hirsutum*; Zm, *Zea mays*; Os, *Oryza sativa*; Ta, *Triticum aestivum*; Td, *Triticum durum*; Sl, *Solanum lycopersicum*; Bn, *Brassica napus*; Pp, *Physcomitrella patens*; Sm, *Selaginella moellendorffii*. Proteins from mono- and dicotyledonous groups are indicated in bold and normal types, respectively. *Picea abies*, *Physcomitrella patens* and *Selaginella moellendorffii* have not been assigned to either group (underlined).

A total of 53 TFs belonging to the HD-ZIP class IV are listed in Table 1. Phylogenetic relationships among individual HD-Zip IV proteins were established using either full length amino acid sequences (Figure 1A), or only HD regions of 43 TFs (Figure 1B). We have investigated phylogeny of TaGL9 from wheat (*Triticum aestivum* L.) in the context of 53 related TFs of the HD-Zip class IV from a range of plant species (Figure 1). Protein sequences were obtained using the BLAST tool from the NCBI database (Table 1). We constructed two types of phylogenetic trees of HD-Zip IV TFs based on either their full-length amino acid sequences (Figure 1A) or their HD domains (Figure 1B). The two types of trees using full-length and HD domain sequences were constructed to understand, which part of sequences (or domains) succumbed to selective pressures. The comparative analyses of these trees indicated that TFs from mono- and dicotyledonous species (mono- and di-cotyledonous species are indicated in bold and normal types, respectively) may have evolved independently, as distributions of TFs in individual sub-branches in both trees segregated. This observation suggested that a divergence between the monocotyledonous and dicotyledonous *HD-Zip IV* genes occurred early during evolution in both types of plants. It is as yet unclear, whether there are any major functional differences between the dicotyledonous and monocotyledonous HD-Zip IV proteins.

**Figure 2:**
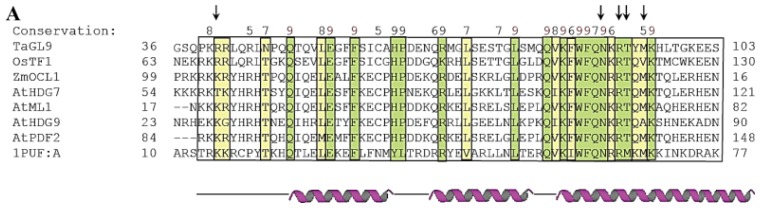

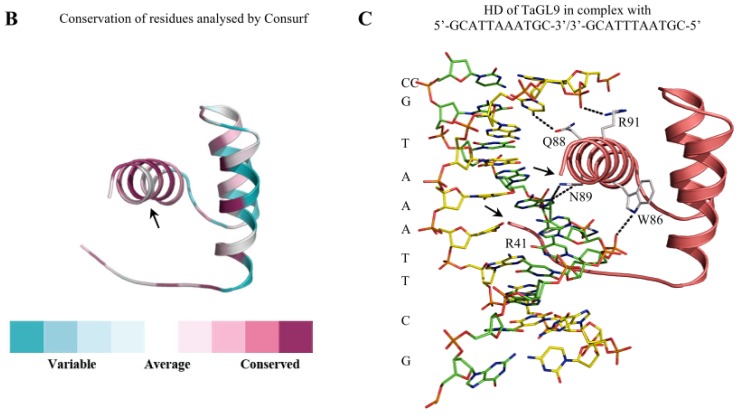
Molecular modelling of the homeobox domain (HD) of TaGL9 from wheat in complex with an 11-bp long DNA fragment. TaGL9 has at least six domains as predicted by ProDom [56]. (**A**) A multiple sequence alignment of selected HD sequences using ProMals3D [51]. The predicted secondary structures are shown in magenta (α-helices) and black (loops). Conservation of residues on a scale of 9–5 is shown at the top of the diagram. The absolutely conserved and similar residues are shaded in green and yellow, respectively. The black box indicates the boundaries of HD domains. Vertical arrows above the alignment point to the DNA-interacting residues shown in panel C; (**B**) HD structure of TaGL9 showing the degree of conservation. The coloured model is based on known HD-Zip IV protein sequences found in the Consurf database [52]. 138 sequences were found by CSI-BLAST [53] but only 66 sequences were unique for the Consurf algorithm to perform calculation. Highly conserved amino acids are coloured in deep magenta, while the least conserved and average ones are coloured in cyan and white, respectively. The black arrow indicates the third helix, for which there is a particularly high level of amino acid conservation; (**C**) A molecular model of HD of TaGL9 in complex with 5′-GCATTAAATGC-3′/3′-GCATTTAATGC-5′; the model was constructed as described [57]. HD of Hox-A9 from mouse (Protein Data Bank accession 1puf, chain A), in complex with a 20-bp DNA duplex fragment 5′-ACTCTATGATTTACGACGCT-3′ [50] served as a template. Ribbon representation in salmon shows the disposition of secondary structure elements. Here, the α-helix 3 (perpendicular to the viewer’s plane) carries most of the residues that mediate contacts between HD and the DNA fragment. The duplex DNA is shown in cpk-green (coding strand) and cpk-yellow (complementary strand). The nucleotides interacting with HD are represented as cpk sticks. The left and right black arrows point to the NH_2_- and COOH-termini of HD, respectively. Separations of ≥3.5 Å between the contacting residues (1-letter codes) of HD and a DNA strand are indicated by black dotted lines. The interplay of the interacting residues within HD suggests that structural rigidity and/or flexibility could impact upon selectivity of DNA binding. It is of note that mainly the TAAA and GCAT segments of the coding and complementary strands, respectively, are interacting with the five highly conserved residues of HD. The nucleotide sequence of the DNA coding strand is shown on the left.

The cDNA of HD-Zip IV gene *GLABRA2-like9* (*TaGL9*) was isolated from a wheat grain cDNA library based on strong interaction of the gene product with the bait sequence 5′-GCATTAAATGC-3′, which contains the L1 box [45]. To have a better understanding on how the HD region of HD-Zip IV proteins interacts with the L1 box, a homology molecular model of the TaGL9 HD binding the 5′-GCATTAAATGC-3′ element was constructed (Figure 2). Chain A of HD from mouse Hox-A9 along with a 20 bp-DNA duplex [50], were used as templates to generate the model. A multiple sequence alignment was performed using ProMals3D [51], and selected HDs from HD-Zip IV subfamily members (Figure 2A) including TaGL9 HD were used to assess conservation of amino acid residues in HD of TaGL9. A total of 138 publicly available amino acid sequences with HDs were found through ConSurf analysis [52] by CSI-BLAST [53] and 66 unique amino acid sequences of the HD-Zip IV proteins including TaGL9 from *Triticum aestivum* (Acc. No. AEI99592) were selected for analysis [52,54]. The ConSurf analysis revealed that among the three α-helices of HD, helix 3 is the region with the highest level of conservation (Figure 2B). This may be because the amino acid residues in the third helix (here designated as the recognition helix) are responsible for most interactions between the TaGL9 HD and the specific DNA element, while the other two helices are most probably responsible for the formation of the hydrophobic core, enabling the recognition helix to achieve an optimal position for formation of the protein-DNA complex [55].

The results of HD sequence analysis by ProMals3D software (Figure 2A) and the model of the TaGL9 HD, which was assigned according to the conservation values of the ConSurf Server (Figure 2B), revealed a group of five amino acid residues that are highly conserved within HDs of all known HD-Zip IV proteins. These five amino acid residues are responsible for the interaction between the HD-Zip IV protein and the 5′-GCATTAAATGC-3′ *cis*-element. The residues contacting DNA are indicated by black dotted lines in Figure 2C. Four of the DNA-interacting residues (Trp86, Gln88, Asn89 and Arg91, Figure 2C) were found to be located within the recognition helix and had an evolutionary conservation score of 9.9. One other amino acid residue (Arg41) was positioned at the *N*-terminal part of HD of TaGL9 and had an evolutionary conservation score of 8. Notably, one amino acid residue (Met93) located in the recognition helix had a relatively low evolutionary conservation score of 6 that indicates a high variability of this residue across HD-Zip IV members (Figure 2B). Nevertheless, results of the ConSurf analysis demonstrated that this Met93 can only be substituted by residues with hydrophobic side chains such as tryptophan, leucine and isoleucine. These data indicate that the specific residue in this position may be important for maintaining the structural integrity of HD or might be involved in protein-protein interaction, as residues with hydrophobic side chains are rarely exposed to a hydrophilic environment.

Comparisons of HD sequences of HD-Zip IV proteins with those of other HD-Zip classes using the homology model of TaGL9 HD revealed a low level of similarity between these sequences (28% sequence identity between either HD-Zip I or II and HD-Zip IV, and 29% sequence identity between HD-Zip III and IV). Further analysis using multiple sequence alignment generated with CLUSTALX [47] showed that the third helix of HD defines the identity of HD domains in HD-Zip TFs from different subfamilies (data not shown). These results indicated that the other two-helices, which contain highly variable amino acid residues, might be responsible for recognition of subfamily-specific *cis*-elements.

The second conserved domain characteristic of HD-Zip IV TFs is a leucine zipper (Zip), which is specific for the HD-Zip IV structure and in this subfamily of HD-ZIP TFs it has been described as a bipartite dimerization leucine zipper-loop-zipper (ZLZ) motif [31]. The motif is located immediately after the third helix of HD. Despite disruption of the leucine zipper of the ZLZ motif by an inserted loop sequence, dimerization via this motif can still occur [31]. Dimerisation is a prerequisite for DNA binding by HD-Zip IV proteins. Unlike other HD proteins, which can bind DNA as monomers, HD-Zip IV TFs can bind DNA efficiently only as either homo- or hetero-dimers [58].

The third domain which is present in all HD-Zip IV proteins is a START domain, which is composed of approximately 200 amino acids residues. In animals, the START domain has been found in proteins belonging to several families including the phosphatidylcholine transfer protein (PCTP), metastatic lymph node 64 (MLN64) and the StAR-related lipid transfer protein 4 (STARD4), but it has not been found to be associated with a DNA binding domain of animal TFs [13,59]. The main role of the START domain in mammals is in binding and transportation of lipid and/or sterol molecules. Approximately three quarters of *Arabidopsis* (26/35) and rice (22/29) proteins with a START domain contain additional protein domain(s) [13]. Among the additional domains are HD, Plekstrin Homology (PH) domain and a domain 1336 (DUF1336) with an as yet unknown function. Similarity of structural features of the START domain-containing HD-Zip IV proteins, and mammalian nuclear receptors containing DNA-binding Zinc fingers and sterol/lipid binding Ligand Binding Domain (LBD) suggest that the START domain in plants might be involved in signal transduction and direct regulation of transcription by binding and transporting steroid-type phytohormones and/or other lipid molecules [13]. The binding affinity of TFs containing a START domain to specific DNA elements may be affected either by a direct protein-lipid/sterol interaction or by an interaction of lipid/sterol with a partner protein, which is bound to the same promoter region. The function of the HD-Zip IV protein HD-GLABRA2 (HD-GL2) from *Arabidopsis* as a negative regulator in phospholipid signalling in roots supports the possible involvement of the START domain in such regulation [26]. It was suggested that binding of lipids by the START domain might be necessary for nuclear transport of HD-Zip IV TFs in a way similar to the model proposed for nuclear transport of the glucocorticoid receptors in mammals [60]. A nuclear localisation of the HD-GL2 protein and the maize HD-Zip IV protein ZmOCL1, have been demonstrated [61,62]. In addition, it has been shown that AD of the maize HD-Zip IV protein ZmOCL1 is located at the NH_2_-terminal region of the START domain [62].

One more distinguishable feature of HD-Zip IV proteins is a conserved HD-START-associated domain (HD-SAD) [10]. Little is known about functions of this domain. However, simultaneous presence of both the START and HD-SAD domains of cotton HD-Zip IV TF GbML1 is required for binding to the *C*-terminal domain of GbMYB25, a key regulator of cotton fibre initiation. This was demonstrated in the EMSA and the yeast 2-hybrid (Y2H) assay using truncated proteins and proteins with point mutations [63].

Using the maize Outer Cell Layer 1 (ZmOCL1) protein as bait in a Y2H screen, the SWITCH complex protein 3C1 (SWI3C1) was identified and was confirmed as an interacting partner *in planta*[62]. However, the domain/motif of ZmOCL1 responsible for this interaction was not mapped. SWI3C1 is a member of the SWI3 protein family and belongs to the C subfamily of such proteins in plants. It possesses all domain characteristics which are found in similar components of the SWITCH/SUCROSE NONFERMENTING (SWI/SNF) ATP-dependent chromatin re-modelling complex of yeast and mammalian origins [64]. ATP-dependent chromatin re-modelling complexes, such as SWI/SNF, temporarily disable histone interactions with DNA, allowing accessibility of DNA to TFs. However, the SWI/SNF complex binds DNA in a non-specific manner. It was shown that the recruitment of this complex in mammals and yeast requires interaction with transcriptional activators [65,66]. In maize, it is likely that ZmOCL1 specifies recruitment of the SWI/SNF complex to a particular promoter region(s), and that its association with this complex facilitates access to specific *cis*-element(s) [40].

The HD-Zip IV proteins are among the longest TFs in plants and have a complex domain structure. Unfortunately, attempts to understand function of these TFs through structural studies have so far produced little data.

## 3. Regulation of *HD-Zip IV* Gene Expression

Our understanding of cell-specific regulation of *HD-Zip IV* gene expression is very limited. In most reports expression of *HD-Zip IV* genes has been observed in outer cell layers, preferentially in the epidermis of developing embryo and/or other plant organs [24,40], including the endosperm, scutellum and roots [40]. The L1 box is a DNA element which directs specific gene expression in epidermal cells [25]. The promoter sequence of only one *HD-Zip IV* gene, *AtML1*, has so far been studied [67]. *AtML1* expression is initially observed in the apical daughter cell of the zygote and later in epidermal cells throughout embryo and plant development [68]. A 101 bp-long sequence containing the L1 box and a putative WUSCHEL-binding site was identified using a *GFP* reporter gene [67]. This sequence was sufficient for embryo-specific activation of the *AtML1* promoter. Interestingly, the L1 box alone was not able to activate expression of the reporter gene. However, several other promoter fragments could activate the *AtML1* promoter in some but not in all epidermal cells. It was concluded that the *AtML1* promoter has a complex modular structure and that several regions of the promoter are involved in spatial and temporal regulation of *AtML1* expression during embryogenesis [67]. The presence of the L1 box was identified in several other *HD-Zip IV* promoters that might suggest feed-back regulation of *HD-Zip IV* genes by their own gene products or by other members of the subfamily [25,26].

Attempts were made to explain cell-specific expression of the *HD-Zip* genes by microRNA (miRNA) involvement. Most miRNAs found in *Arabidopsis* are targeted to TF genes, which are involved in plant development and particularly in development of shoot and floral meristems. One of the well characterised cases of miRNA regulation in plants is recognition of a conserved region at the beginning of the START domain in the HD-Zip III subfamily of TFs, by miR165/166 [69]. Because of tight high similarity of domain structures between HD-Zip III and HD-Zip IV proteins, this regulatory system may also be applied to *HD-Zip IV* genes. Regulation of *HD-Zip IV* genes by miRNAs might be supported by the presence of a conserved 17-nucleotide motif located at in the 3′UTR region of the *HD-Zip IV* genes. This conserved sequence was found in *PaHB1*, *PaHB2* and several other *HD-Zip IV* genes from *Arabidopsis*, sunflower and cotton [35]. However, alternative mechanisms, which could involve the 17 bp long motif in regulation of gene expression, may also exist. Some metazoan *HB* genes expressed during embryogenesis contain regulatory elements located in their 3′-UTR regions that influence subcellular localisation, translation and stability of mRNAs [35]. Therefore, further investigation is required to test whether the conserved motif in *HD-Zip IV* genes has any regulatory function involving miRNAs.

Expression of the *FWA*/*HDG6* gene was found to be controlled by epigenetic hyper-methylation of two direct repeats situated at the 5′ region of the gene around its transcription start site [70]. Ectopic *FWA* expression led to a late-flowering phenotype, which resulted from an in inhibition of function of the *FLOWERING Locus T.* (*FT*) gene by the *FWA* gene product in vegetative tissue of *Arabidopsis*[70–72]. It was demonstrated that FWA expression is imprinted in endosperm [30,73]. *FWA* is silenced by DNA methylation in vegetative tissue but it is de-methylated in the central cell of the ovule and after fertilisation *FWA* is expressed in the endosperm from the maternal copy of the gene [74].

## 4. Roles of HD-Zip IV TFs

### 4.1. Development and Maintenance of Epidermal Cell Layers

Expression of genes encoding the HD-Zip subfamily IV proteins can typically be observed in the outer cells of embryos and in the epidermal or sub-epidermal layers of developing plant organs, where regulation of epidermal cell fate, trichome formation and anthocyanin accumulation occur [32,33,75]. The epidermis plays a critical role in plant defence against pathogens and in protection from environmental stresses [23]. Differentiation of the epidermis is one of the major events in plant development. During embryogenesis the basic plant body plan is realised by specifying position of shoot and root meristems. The meristems contain groups of stem cells, which provide initial material for the development of plant organs after seed germination. During meristem formation protoderm differentiation leads to organisation of inner and outer cell layers [76]. Epidermal-specific expression patterns of the *HD-Zip IV* genes have been observed in a variety of plant species including *Arabidopsis*[24], rice (*Oryza sativa*) [41], maize (*Zea mays*) [38], cotton (*Gossypium hirsutum*) [36] and Norway Spruce (*Picea abies*) [34].

A total of sixteen HD genes, (*HDG*s) namely *HDG1*–*HDG6*/*FWA*, *HDG7*–*HDG12*, *GLABRA2* (*GL2*), *ANTHOCYANINLESS2* (*ANL2*), *AtML1*, and *PDF2*, comprise the *Arabidopsis* HD-Zip IV subfamily [24]. At least some of these genes were shown to be involved in cell fate determination of the epidermal cell layer by regulating cell layer-specific gene expression (Table 1). *GL2* or *AtHB-10* from *Arabidopsis* was the first identified member of the HD-Zip IV subfamily. The expression of *GL2* was detected only in the specific epidermal cells responsible for formation of trichomes and root hairless epidermal cells. Absence of trichomes was observed in the *gl2* mutant suggesting that the *GL2* gene may play a role in trichome formation [77]. In addition, the *gl2* mutation caused root-hair formation from hairless epidermal cells, but other cellular processes that usually take place during differentiation of root-hairs and hairless cell files were not affected [78]. *Arabidopsis HDG11* and *HDG12* genes were reported to be involved in trichome branching. The *hdg11*, but not the *hdg12*, mutant had more branched trichomes than wild-type plants. However, the phenotype of the *hdg11* mutant was enhanced in double mutants of *hdg11* and *hdg12*[24]. Surprisingly, the characteristic phenotype of *gl2* mutant was not altered in the *gl2 hdg11* and *gl2 hdg12* double mutants [24].

Involvement of *HD-Zip IV* genes in differentiation of the epidermis has been clearly demonstrated using mutants of two other *HD-Zip IV* genes from *Arabidopsis*[33]. Single gene knockout mutants of *PDF2* and *ATML1*, which have a high level of sequence identity, retained normal shoot development, while the double mutant had severe defects in shoot epidermal cell differentiation. This suggests that *PDF2* and *ATML1* have the same or very similar functions in differentiation of epidermal cells, which are possibly realised by interacting with L1 boxes of downstream target-gene promoters [33].

### 4.2. Development of Sub-Epidermal Cell Layers and Control of Anthocyanin Pigmentation

The product of the *HD-Zip IV* gene *ANL2* from *Arabidopsis* shares a high level of amino acid sequence similarity with the product of the *AtML1* gene, but has different function. ANL2 controls anthocyanin pigmentation of the leaf sub-epidermal layer and cellular organisation of the primary root [32]. Plant anthocyanins are pigments that are responsible for a range of colours such as red, blue and purple. The accumulation of anthocyanins occurs during specific plant developmental stages and only in certain plant tissues. The accumulation of anthocyanins is also induced by environmental stimuli such as light, temperature, nutrients and by different stresses [32]. It is interesting that *ANL2* controls pigmentation of leaves and stems, but is not involved in regulation of anthocyanin synthesis in the internal layer of the seed coat. The *anl2* mutant has an abnormal radial root patterning. This mutant produces extra cells known as intervening cells located between the cortical and epidermal layers [32]. Based on these data it was proposed that *ANL2* might be involved in the maintenance of sub-epidermal layer identity during plant development and that it controls the process of anthocyanin accumulation in sub-epidermal cells.

### 4.3. Drought Tolerance

The up-regulation of the *Arabidopsis HD-Zip IV* gene *HDG11* was found to contribute to increased drought tolerance [17]. Such observation originates from characterisation of the *enhanced drought tolerance 1* (*edt1*) mutant, which demonstrated better drought tolerance characteristics than control plants. The mutant had dramatically increased expression levels of the *HDG11* gene. The mutant phenotype included improved root architecture, a more extensive root system, and reduced stomatal density. The mutants also had higher levels of abscisic acid, which further expanded the capacity for stress response. In addition higher levels of Superoxide Dismutase (SOD) activity and Pro in mutant plants could potentially contribute to better osmotic adjustment and Radical Oxygen Species (ROS) detoxification. The same phenotype was observed when *HDG11* was constitutively over-expressed in wild-type *Arabidopsis*. The authors suggested that such elevated levels of expression resulted in significantly increased amounts of HDG11 protein in the nucleus, which made it a dominant TF that successfully competed for any available HD-binding site in the genome [17]. No changes in epidermal cell layers or cuticle development were reported [17]. However, higher levels of ABA in the *edt1* mutant suggested that a mechanism of drought response regulation by HDG11 might be similar to that of the members of the HD-Zip I subfamily [21,22].

### 4.4. Function of HD-Zip IV Genes in Commercially Important Plant Species

Data obtained with *Arabidopsis* on the regulation of trichome development by *HD-Zip IV* genes, stimulated attempts to understand roles of these genes in agriculturally important plants with the aim to apply the gained knowledge for improvement of important traits. Cotton (*Gossypium* spp.) fibres, which are also known as seed trichomes, have a high economic value. Therefore, an understanding of the molecular mechanisms responsible for fibre initiation and control of development is important for enhancing yield and quality of fibres [37]. Cotton fibres are distinct from the *Arabidopsis* trichomes. They are exceptionally elongated, usually not branched and when mature, composed of almost pure cellulose [36]. The *Gossypium arboretum* L. *HOMEOBOX* genes 1 and 2 (*GaHOX1* and *GaHOX2*) are cotton genes encoding HD-Zip IV TFs. *GaHOX1* was found to be the closest homologue of *GL2*, while *GaHOX2* was more similar to *AtML1* and *PDF2* from *Arabidopsis*. Functional complementation with the *GaHOX1* gene was observed when it was expressed under the *GL2* promoter in the *Arabidopsis gl2* mutant. This suggested that the GaHOX1 protein has the same function as GL2 in regulation of trichome development. In contrast, *GaHOX2* might be involved in seed coat development rather than in trichome growth regulation [36].

Expression levels of the *HD-Zip IV* gene from *Gossypium hirsutum* L., known as *HOMEODOMAIN 1* (*GhHD-1*) were lower in the ovule epidermis of a fibreless mutant compared to those expressed in the ovule epidermis of wild-type plants [79]. *GhHD-1* was predominantly expressed in epidermal and sub-epidermal layers of cells. The fact that this gene was up-regulated during conversion of cells into fibres might suggest its involvement in fibre differentiation [37]. A close homologue of *GhHD-1* from *G. barbadense* (*GbML1*) has been shown to interact with GhMYB25 in the Y2H assay. The GhMYB25 protein in turn was demonstrated to be involved in the activation of the fibre-preferential cotton gene *RD22-like1* (*RDL1*) by direct binding to its promoter region, and hence this gene also has a role in fibre development [63]. Because a large number of MYBs are expressed in cotton ovules, the authors speculated that GhHD-1 might interact with various MYB factors in different cell types to enable diverse functions [80]. Phenotyping of transgenic plants with silenced *GhMYB25* and *GhHD-1* genes indicated that these genes work in cooperation. Both types of transgenic plants had reduced trichome numbers and a delayed initiation of seed fibre differentiation. However, some differences in function of *GhMYB25* and *GhHD-1* were also found. For example, a decrease of fibre length was observed only in lines with silenced *GhMYB25* but not in the *GhHD-1*-silenced lines [37,81].

A total of seventeen HD-Zip IV genes, also known as *Outer Cell Layer* (*OCL*) genes, have been identified in the maize genome. However, only a few of them have been characterised in detail (Table 1). Most are preferentially expressed in immature reproductive organs and/or the epidermal (L1) cell layer [40]. It was shown that *ZmOCL1*, *ZmOCL3*, *ZmOCL4* and *ZmOCL5* have epidermis-specific expression patterns, whereas expression of *ZmOCL2* was restricted to the sub-epidermal layer (L2) [38,82]. It was demonstrated that *ZmOCL4* is involved in anther and trichome (macrohairs in maize) development [39]. The *ocl4* mutant has ectopic growth of macrohairs. Surprisingly, expression of *ZmOCL4* did not rescue the *gl2* mutant in *Arabidopsis*, and even enhanced the influence of the *gl2* mutation on trichome development. The *ocl4* mutant also has partial male sterility. Smaller size of anthers in mutant maize plants compared to the wild-type plants and absence of anthocyanins suggested a premature termination of anther development in the mutant [39].

Another maize gene, *ZmOCL1*, was found to have a role in regulation of kernel development in maize. A reduction in kernel size was observed in transgenic maize plants transformed with a construct for expression of a fusion of the repressor domain of the *Drosophila* Engrailed protein and the dimerisation domains of ZmOCL1, which converted ZmOCL1 from an activator to a repressor [7].

Analysis of the rice *HD-Zip IV* genes revealed that while some of these are involved in regulation of processes that take place in the epidermis, the function of others seems to be different. Nine GL2-type *Rice outermost cell-specific* (*Roc*) genes were found in the rice genome using a BLAST search [42]. Five of these genes were isolated as full length cDNA clones and designated *Roc1* to *Roc5* (Table 1). Expression of all five genes was restricted to the leaf epidermis [41,42]. Specific expression of *Roc1* begins in the outermost cells, and precedes protoderm differentiation. The authors suggested two possible explanations for this phenomenon: (1) epidermal cell fate is dependent on *Roc1* expression; or (2) *Roc1* expression occurs before the determination of epidermal cell fate [41,42]. Expression of *Roc1* was also detected in the outermost cell layer of callus before organogenesis, and was independent of whether the cells did or did not differentiate. Wounding by cutting has induced *Roc1* expression in newly formed outer layers. These observations suggested that *Roc1* is expressed in a position-dependent manner and that *Roc1* is not involved in determination of fate of epidermal cells [41].

Another rice gene, *Roc5*, has been recently shown to be associated with development and formation of bulliform cells in rice leaves [83]. The bulliform cells act as specialised epidermal cells that are involved in protection against pathogen invasions and in prevention of water loss during stress. These cells are found on the adaxial surface of leaves in all monocotyledous plants except *Helobiae*[84,85]. *Roc5* overexpression and suppression in transgenic rice plants were used to determine how *Roc5* affects development of bulliform cells in rice. Overexpression of the *Roc5* gene resulted in a decrease in bulliform cell number and size that in turn lead to the adaxial rolling of leaves. On the contrary, suppression of *Roc5* in transgenic rice led to an increased number and size of bulliform cells and the abaxial rolling of leaves. These data indicate that *Roc5* is a negative regulator of bulliform cell development and bulliform cell-dependent leaf rolling [83]. It would be also interesting to investigate the role of *Roc2* in bulliform cell development since physical interaction of *Roc5* and *Roc2* has been demonstrated to occur in yeast [42].

*Rice Transcription Factor 1* (*OsTF1*) is a rice *HD-Zip IV* gene, which shares a relatively low sequence similarity with *AtML1* and *ZmOCL5*[86]. HD of OsTF1 is located at the *N*-terminal part of the protein, unlike HDs in most other HD-Zip IV members, which are situated either in the middle or at the *C*-terminal part of the protein [86]. However, OsTF1 contains all domains and motifs that are characteristic of the HD-Zip IV proteins [68]. Regardless of the low level of similarity between protein sequences of OsTF1 and AtML1 or ZmOCL5, the epidermis-specific expression pattern of *OsTF1* in the early embryo is consistent with the expression pattern of other *HD-Zip IV* genes. The precise role of the *OsTF1* gene remains unclear, but it may contribute to regulation of organ or tissue differentiation in the developing embryo. High levels of expression of this gene were observed shortly before organ differentiation occurred [86].

The closest wheat homologue of *OsTF1*, *TaGL9*, was found to be expressed in embryo epidermis and adjacent to the embryo portion of the syncytial endosperm during early grain development. However, later in development *TaGL9* expression relocated to the main vascular bundle of the scutellum. Such patterns of expression suggest that *TaGL9* could play a role in development and maintenance of inner surface layers of the vascular bundle [45]. A gene encoding a lipid transfer protein with the same pattern of expression has been identified in wheat [87]. This gene may be a potential target gene of *TaGL9*, or it may be that expression of both genes is regulated in a similar way by other TF(s).

### 4.5. Regulation of Cuticle Formation and Plant Protection

Recently, a new function of HD-Zip IV TFs has been described in the context of regulation of biosynthesis and transport of plant cuticle components. Plant cuticle has an important role in establishment of a protective hydrophobic layer on the external cell wall of epidermal cells in aerial parts of plants [88]. The cuticle layer provides protection to plants from biotic and abiotic stresses through its specific chemical, physical and optical properties [23,89,90]. Thickness, chemical composition and structure of a cuticular layer, as well as environmental parameters such as temperature at the leaf surface, are important determinants of cuticle permeability [89,91,92].

The regulation of activity of genes involved in cuticle biosynthesis is still poorly understood. Members of two families of TFs have been demonstrated to control cuticle biosynthesis and accumulation of cuticular wax during plant development and during plant responses to stresses. These TFs are the ethylene responsive factors (ERF-type transcriptional regulators) WAX INDUCER1/SHINE1 (WIN1/SHN1) and WAX PRODUCTION1 (WPX1), and several MYB TFs [93–97]. Recently, a new regulatory pathway controlling cuticle development was reported. It involves a *Curly Flag Leaf1* (*CFL1*) gene, which encodes a protein containing a WW domain [29]. As the name suggests, the WW domain contains two conserved tryptophans, which can bind proline-rich peptides [98]. Overexpression of both rice and *Arabidopsis CFL1* in transgenic *Arabidopsis* plants showed severely impaired cuticle development. It has been demonstrated that AtCFL1 interacts directly with the HD-Zip IV protein HDG1, which negatively regulates its activity [29]. It was also recently demonstrated that HDG1 contains an *N*-terminal Ethylene-responsive element binding factor-associated Amphiphilic Repression (EAR) motif [99]. Transcriptional activators with attached EAR motifs usually function as dominant repressors [100]. However, over-expression of *HDG1* in wild-type *Arabidopsis* did not produce a phenotype similar to those observed either during over-expression of *AtCFL1* or repression of *HDG1*. It was speculated that the six amino acid residue EAR motif of HDG1 is too short to act as a dominant repressor without assistance from other cofactors [99].

Recently, it has been shown that transgenic maize with the overexpressed *HD-Zip IV* gene *ZmOCL1* had altered wax composition in juvenile leaves [101]. This finding was supported by the fact that *ZmOCL1* regulates expression of several genes involved in lipid transport and metabolism, and potentially could be involved in cuticle biosynthesis and deposition [101]. Based on microarray analysis, 14 genes were identified to be directly or indirectly regulated by over-expression of the *ZmOCL1* gene. Half of these genes encode proteins that are known to be involved in biosynthesis and/or transport of lipid molecules and plant defence. Among them are the type 2 lipid transfer protein (nsLTPII), an AtCXE-18 carboxylesterase, a SEC14/phosphatidylinositol transport protein (PITP), three ABC transporters (WBC11/ABCG11-like) and a fatty acid reductase (FAR). FAR is closely related to the *Arabidopsis* ECERIFERUM 4 (CER4) protein, which is responsible for synthesis of long chain primary alcohols from fatty acid precursors [102].

The major function of carboxylesterase enzymes is to hydrolyse esters of short-chain fatty acids [103]. However, the majority of carboxylesterases are associated with plant defence [104]. The SEC14/PITP transport proteins, similar to the one activated by *ZmOCL1*, are known to be involved in *trans*-Golgi export pathways [105] and therefore can be potentially involved in biosynthesis of cuticular lipids. SEC14/PITP can catalyse phosphatidylinositol and phosphatidylcholine transfer *in vitro*. In mammals, several diseases develop due to deficiencies of PITPs [106].

As mentioned above, another group of genes activated by *ZmOCL1* encode ABC transporters. The ABC transporters belonging to the White Brown Complex (WBC) family are specialised in ATP-dependent translocation of steroids and other lipid molecules in mammals [107]. The analysis of *Arabidopsis* mutants has shown that the ECERIFERUM 5 (CER5) protein WBC12/ABCG12 and CER5-like protein WBC11/ABCG11 are key components of cutin and wax transport from their site of biosynthesis to the cuticle layer [108,109]. It is known that *CER5* is a stress-responsive gene and that it is up-regulated by the stress hormone ABA, drought and high salinity [90].

The gene encoding type 2 lipid transfer protein (nsLTPII) is among genes regulated by HD-Zip IV TF *ZmOCL1*. It has been proposed that LTPs play an essential role in transport of cuticular lipids through plant cell walls [110]. Recently the role of LTPs in the export of cuticular components was confirmed by analysis of *Arabidopsis* plants with a mutation in the *LTPG1* gene. The *ltpg1* mutant had a thinner wax layer; this finding suggested a role of LTPG1 in facilitation of the export of wax components through the plasma membrane [111,112]. Some NsLTPs have been shown to have a role in plant protection against fungal infection and insects [113,114]. It is also known that over-expression of HvLTP2 in barley increases tolerance to *Pseudomonas syringae*[115], while the *ltpg1* mutant showed increased susceptibility to fungal infections [112].

Plant defencins (γ-thyonines) comprise an LTP subfamily known to have strong antifungal and antibacterial properties [116–118]. A group of wheat genes encoding defensins was found to be specifically expressed in the outer cell layers of developing grain [119]. It has been recently demonstrated that a wounding-inducible HD-Zip IV TF, which is preferentially expressed in wheat grain, can activate promoters of several grain-specific defensins in a transient expression assay (our unpublished results). This findings support data [101] on activation of the LTP gene by *ZmOCL1* over-expression, and suggests that HD-Zip IV TFs can be involved in plant protection from pathogens either directly, by activation of certain pathogenesis-related proteins, or indirectly, by activation of proteins that are involved in cuticle formation.

## 5. Conclusions and Future Perspectives

The existing data regarding HD-Zip IV TFs suggest their involvement in regulation of development and maintenance of outer cell layers of plant organs, which may play an important role in plant defence from pathogens and protection from abiotic stresses. Emerging data imply that the HD-Zip IV proteins regulate cuticle biosynthesis and wax accumulation. This function bears similarity to functions of earlier discovered TFs from the ERF and MYB families. The inducibility of cuticle-related ERF and MYB TFs by ABA, drought and high salinity has been reported and ability of these TFs to confer stress tolerance to transgenic *Arabidopsis*, alfalfa and rice plants has been demonstrated [93–96]. It would be interesting to examine if any of the *HD-Zip IV* genes are involved in abiotic stress responses by modulation of size and content of cuticle.

Also fascinating is the fact that some *HD-Zip IV* genes were found to be induced by wounding and could regulate some pathogen responsive genes. Further studies are required to determine if ‘clever’ up-regulation of *HD-Zip IV* genes in transgenic plants can directly or indirectly enhance plant defence. Such ‘clever’ up-regulation would entail the use of outer cell layer-specific promoters, or modification of the promoters of *HD-Zip IV* genes themselves by the addition of enhancer sequences.

In contrast to *Arabidopsis*, where many *HD-Zip IV* genes are expressed in vegetative tissues, in grasses these genes are expressed mostly in reproductive parts of the plant. A role for *ZmOCL1* in maize kernel development has been demonstrated [7]. Further comprehensive analysis of function needs to be done for other cereal HD-Zip IV family members, which are predominantly expressed in flowers and grain.

The mechanism of *HD-Zip IV* promoter regulation remains unclear and requires further attention. Nevertheless, *HD-Zip IV* genes are a promising source of tissue-specific promoters of moderate strength, particularly for specific expression in epidermal and/or sub-epidermal layers, as well as in specific parts of flowers and grain.

## Figures and Tables

**Table 1:** Published *HD-Zip IV* genes and their characteristics.

Plant	Gene name	Accession	Tissue localisation	Suggested function*	Cited
*Arabidopsis thaliana*	*HDG1*	NP_191674	In trichomes at the base of young leaf, endodermal cell lines around emergent lateral root, stamen filament	Cuticle development	[24,29]
*Arabidopsis thaliana*	*HDG2*	NP_172015	Outer cell layers of shoot apical meristems, early flower primordia, nucellus, seed coat, epidermal layers of young leaves, hairless cell files of hypocotyl epidermis, primary root tips	Embryo development	[24]
*Arabidopsis thaliana*	*HDG3*	NP_180796	Siliques and seedlings	Cotyledon development	[24]
*Arabidopsis thaliana*	*HDG4*	NP_193506	Flowers	Flower organ development	[24]
*Arabidopsis thaliana*	*HDG5*	NP_199499	All plant organs except root, outer cell layers of shoot apical meristems, early flower primordia, nucellus, epidermis of the stamen filament, stomatal guard cells of the carpel	Not determined	[24]
*Arabidopsis thaliana*	*FWA/HDG6*	NP_567722	Endosperm	Endosperm development	[30]
*Arabidopsis thaliana*	*HDG7*	NP_200030	Base of leaf primordia, apical region of the heart-stage embryo, lateral root primordia and tips, seedlings	Not determined	[24]
*Arabidopsis thaliana*	*HDG8*	NP_186976	Endosperm and embryo at early stages of development, after flower organ development	Flower, seed development	[24]
*Arabidopsis thaliana*	*HDG9*	NP_197234	Developed flowers, chalazal, embryo sac	Embryonic development	[24]
*Arabidopsis thaliana*	*HDG12*	NP_564041	Apical meristem, stamen filament, nucellus, embryo, young epidermal tissues, lateral root tip	Trichome development	[24]
*Arabidopsis thaliana*	*GL2*	NP_565223	Epidermis of leaves and roots, developing trichomes and surrounding epidermal cells, hairless cell files of hypocotyl epidermis	Trichome and root hair development	[31]
*Arabidopsis thaliana*	*ANL2*	NP_567183	Leaves, stems, buds, roots	Anthocyanin distribution and root development	[32]
*Arabidopsis thaliana*	*ATML1*	NP_193906	Flower bud, lower parts of siliques	Shoot epidermal cell differentiation	[33]
*Arabidopsis thaliana*	*PDF2*	NP_567274	Flower buds, shoot apices	Shoot epidermal cell differentiation, cotyledon development	[33]
*Picea abies*	*PaHB1*	AAG43405	Embryo	Embryo development	[34]
*Picea abies*	*PaHB2*	AAL83725	Embryo	Embryo development	[35]
*Gossypium spp.*	*GaHOX1*	ABY41242	Fibre cells	Cotton fibre development	[36]
*Gossypium spp.*	*GaHOX2*	ABY67263	Fibre cells at early developmental stages	Seed coat development	[36]
*Gossypium hirsutum*	*GhHD-1A*	AFO11041	Epidermal cells	Epidermal cell determination	[37]
*Gossypium hirsutum*	*GhHD-1D*	AFO11042	Epidermal cells	Epidermal cell determination	[37]
*Zea mays*	*ZmOCL1*	CAG38614	Immature ears, epidermis specific expression in embryo, meristems, young organ primordia, juvenile leaves	Kernel development, cuticle deposition	[7]
*Zea mays*	*ZmOCL2*	CAB96422	Apical meristem, sub-epidermal layer of floral meristems immature ears, young female gametophyte, kernels, root	Not determined	[38]
*Zea mays*	*ZmOCL3*	CAB96423	Developing embryo and endosperm, epidermis specific expression in embryo, meristems, young organ primordial, juvenile leaves	Specification of organ identity	[38]
*Zea mays*	*ZmOCL4*	CAB96424.2	Shoot epidermis, meristems and young organ primordia, immature tassels	Anther and trichome development	[39]
*Zea mays*	*ZmOCL5*	CAB96425	Immature tassels, epidermis specific expression in embryo, meristems, young organ primordia,	Not determined	[40]
*Zea mays*	*ZmOCL6*	DAA34955	Immature tassels, immature ears, epidermal cells of juvenile leaves	Not determined	[40]
*Zea mays*	*ZmOCL7*	DAA34956	Immature ears, epidermal cells	Not determined	[40]
*Zea mays*	*ZmOCL8*	DAA34957	Immature tassels, epidermal cells	Not determined	[40]
*Zea mays*	*ZmOCL9*	DAA34958	Young developing kernels, L2 cells of shoot apical meristem	Not determined	[40]
*Zea mays*	*ZmOCL10*	DAA34959	Immature tassels, epidermal layer of juvenile leaves	Not determined	[40]
*Zea mays*	*ZmOCL11*	DAA34960	Mature tassels that include pollen	Not determined	[40]
*Zea mays*	*ZmOCL12*	DAA34961	Not determined	Not determined	[40]
*Zea mays*	*ZmOCL13*	DAA34962	Immature tassels, epidermis of shoot apical meristem	Not determined	[40]
*Zea mays*	*ZmOCL14*	DAA34963	Immature tassels, epidermal cells	Not determined	[40]
*Zea mays*	*ZmOCL15*	DAA34964	Immature tassels, epidermis of shoot apical meristem	Not determined	[40]
*Zea mays*	*ZmOCL16*	DAA34965	Young developing kernels, epidermal cells	Not determined	[40]
*Zea mays*	*ZmOCL17*	DAA34966	Immature tassels, epidermis of shoot apical meristem	Not determined	[40]
*Oryza sativa*	*OsRoc1*	BAB85750	Shoot apex, inflorescence apex, leaf blade	Embryogenesis	[41]
*Oryza sativa*	*OsRoc2*	BAC77155	Shoot apex, inflorescence apex, leaf blade	Not determined	[42]
*Oryza sativa*	*OsRoc3*	BAC77156	Shoot apex, inflorescence	Not determined	[42]
*Oryza sativa*	*OsRoc4*	BAC77157	Roots	Not determined	[42]
*Oryza sativa*	*OsRoc5*	BAC77158	Leaf epidermis	Epidermal differentiation, bulliform cells development	[42]
*Oryza sativa*	*OsROC6*	Q7Y0V7	Not determined		[42]
*Oryza sativa*	*OsRoc7*	BAC77160	Epidermis	Epidermal differentiation	[42]
*Oryza sativa*	*OsRoc8*	BAC77161	Epidermis	Epidermal differentiation	[42]
*Oryza sativa*	*OsRoc9*	BAC77162	Epidermis	Epidermal differentiation	[42]
*Oryza sativa*	*OsTF1*	Q5ZAY0	Grain	Embryogenesis	[43]
*Oryza sativa*	*OsHOX9*	A2Z8L4	Not determined		[44]
*Triticum aestivum*	*TaGL9*	AEI99592	Grain specific expression	Not determined	[45]
*Triticum durum*	*TdGL9H*	AEI99593	Early embryo, endosperm around embryo, later in scutellar vascular bundle	Maintenance of scutellar vascular bundle	[45]
*Solanum lycopersicum*	*SlCD2*	NP_001234657	Epidermal cell and cuticle	Epidermal cell and cuticle development	[20]
*Physcomitrella patens*	*PpHDZ41*	DAA05775	Not determined	Not determined	[46]
*Selaginella moellendorffii*	*SmHDZ44*	DAA05774	Not determined	Not determined	[46]

* It should be taken in consideration that the current data on HD-Zip IV function are dependent on the available data, and thus in some instances function of HD-Zip IV TFs could be speculative.
